# Nationwide Hypothermic Machine Perfusion for ECD and DCD Kidney Transplantation in Belgium: One-Year Outcomes and Impact on Transplant Rates and Budget Impact Analysis

**DOI:** 10.3389/ti.2025.15282

**Published:** 2025-11-13

**Authors:** Tom Darius, Ina Jochmans, Maxime Foguenne, Eric Hoste, Caren Randon, Bart Bracke, Geert Roeyen, Nicholas Gilbo, Laurent Weekers, Daniel Jacobs-Tulleneers-Thevissen, Karl Martin Wissing, Tineke Bogaerts, Dimitri Mikhalski, jean-Philippe De Wilde, Joël Daems, Jacques Pirenne

**Affiliations:** 1 Department of Surgery, Surgery and Abdominal Transplant Unit, Cliniques Universitaires Saint-Luc, Brussels, Belgium; 2 Department of Surgery, Abdominal Transplant Surgery, UZ Leuven, Leuven, Belgium; 3 Department of Intensive Care Medicine, Universitair Ziekenhuis Gent, Ghent, Belgium; 4 Department of Hepatobiliary, Endocrine, and Transplantation Surgery, Universitair Ziekenhuis Antwerpen, Edegem, Belgium; 5 Division of Abdominal Surgery and Transplantation, CHU de Liege, Liège, Belgium; 6 Department of Surgical Oncology, Thoracic Surgery and Transplantation, Universitair Ziekenhuis Brussel, Brussels, Belgium; 7 Department of Abdominal Surgery and Transplantation, Hopital Erasme, Brussels, Belgium; 8 Rijksinstituut voor Ziekte- en Invaliditeitsverzekering, Brussels, Belgium

**Keywords:** hypothermic machine perfusion, kidney transplantation (KT), hypothermic oxygenated machine perfusion, national implementation, budget impact analysis

## Abstract

In September 2022, Belgium implemented a nationally reimbursed HMP service for all ECD and DCD kidneys procured and transplanted within the country. We retrospectively analyzed data from 242 kidney transplantations preserved with continuous HMP between October 2022 and September 2023. Active oxygenation (HMPO_2_) was applied in DCD donors aged >50 years. One-year outcomes for all HMP kidneys included delayed graft function (DGF) in 14.4%, estimated glomerular filtration rate of 50 mL/min/1.73 m^2^, 10.1% acute rejection, 96.3% death-censored graft survival, and 98.3% patient survival. DGF rates were lower in ECD kidneys (9.1%) and in DCD ≤50 years (9.5%), while higher in DCD >50 years (19.6%). National transplantation rates of DCD kidneys significantly increased from 90 to 175 per year (p < 0.0001), but not for ECD kidneys (from 45 to 54 per year (p = 0.2965) post-HMP implementation without affecting kidney export. The annual cost savings from reduced dialysis requirements were estimated at €3.59 million. The national implementation of a centralized HMP service in Belgium led to excellent one-year transplant outcomes, increased utilization of ECD and DCD kidneys, and substantial healthcare cost savings. These findings support HMP, and where appropriate HMPO_2_, as the new standard of care for kidney preservation in Belgium, with potential implications for broader international collaboration.

## Graphical Abstract

**Figure:**
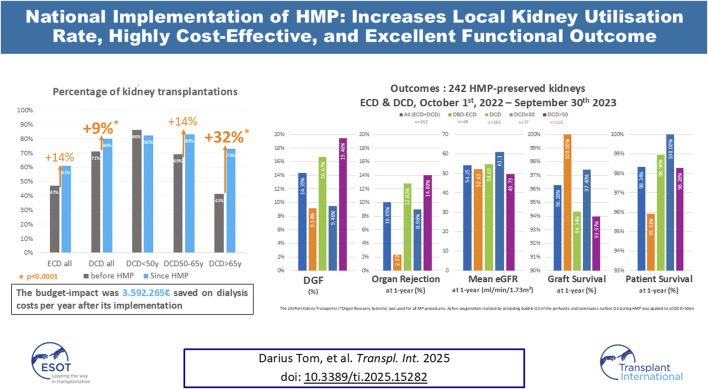

## Introduction

Over the past 2 decades, hypothermic machine perfusion preservation (HMP) has gained traction as a method of donor kidney preservation. Continuous HMP, initiated immediately after procurement and maintained until implantation, has demonstrated superior transplant outcome compared with static cold storage (SCS) and endischemic application [1–6]. It reduces the risk of delayed graft function (DGF), irrespective of donor type and cold ischemia time [7–11]. Only a few publications reported an economic evaluation of the use of HMP as compared with SCS [12–14]. Two reports on the results of Euotransplant Machine Perfusion trial of Moers C et al confirmed cost savings in favor for HMP [12, 13]. One of the main reasons for the cost saving was the lower dialysis costs in the HMP group due to the decreased incidence of DGF.

Despite its proven benefits, only a few European countries (France, Netherlands and Switzerland), have implemented this technique at a national level through structured perfusion services. In Belgium, a major policy shift occurred in June 2021 when the National Institute for Health and Disability Insurance (NIHDI) approved reimbursement for HMP for all kidneys procured and transplanted in Belgium from donors after brain death (DBD) with expanded criteria donors (ECD) and donors after circulatory death (DCD), effective from September 2022. This initiative was initiated in particular to increase the national utilization of ECD and DCD kidneys. A dedicated national kidney HMP group successfully established a centralized national perfusion service (NPS) within a short timeframe.

This study has four primary objectives:

1. Discard Analysis and Feasibility: To assess overall kidney discard reasons and the feasibility of connecting transplantable kidneys to the HMP device.
2. Clinical Outcomes: To evaluate the one-year functional outcomes of kidneys preserved with continuous HMP during the first year of national implementation.
3. Economic Evaluation: To perform a budget impact analysis comparing dialysis costs with the costs associated with HMP.
4. Utilization Impact: To analyze the effect of HMP implementation on the national utilization rate of ECD and DCD kidneys for transplantation in Belgium, covering the period from October 2017 to March 2024.

Additionally, the study outlines the logistical and organizational challenges encountered during the implementation of the national HMP service and discusses future perspectives for its development.

## Materials and Methods

### HMP Reimbursement Criteria, Lump-Sum and Kidney Allocation

The NIHDI approved reimbursement for HMP as preservation technique for all ECD and DCD kidneys that are procured and transplanted in Belgium. ECDs are defined as brain-dead donors aged 50–60 with at least two of the following: arterial hypertension, serum creatinine above 1.5 mg/dL, or death due to a stroke, or any donor over 60 years old. All DCD donors are included regardless of age. The lump-sum of 3500€ per HMP-preserved kidney was calculated on the sum of the cost of the disposables of the HMP device (machine perfusion kit and accessories (connections tubes, canules, sterile drapes), a preservation specialist-fee and a transport from a central hub to the donor centrum and return transport. During the negotiation with the third party providing the HMP infrastructure, it was agreed that at the moment of initiation of this national HMP-program this lump-sum would be sufficient to cover most of the expenses for this project.

All seven Belgian kidney transplant centers agreed to a three-year pilot program (from September 2022 to August 2025) to use HMP as much as possible. HMP was not mandatory and preservation of these kidneys by SCS was allowed.

All kidneys were allocated according to the standard Eurotransplant allocation system with no exceptions.

### Organization of the National Perfusion Service

The centralized NPS consists of a small team of 10 dedicated, well trained, preservation specialists (with a maximum capacity of 3 simultaneous multiorgan procurement procedures) supervised by experienced perfusionists, a central hub in Brussels where 6 machine perfusion devices are stored, and a 24/24h helpdesk. Activation of this service is done by the local donor transplant coordinator, preferably after the kidney is accepted by a Belgian transplantation center and full fitting the reimbursement criteria for HMP and before the start of the procurement procedure. This service consists of a priority transport by car of the preservation specialist and the machine perfusion (MP) device(s) to the donor hospital. The preservation specialist is responsible for the set-up of the MP device(s), support of the procurement surgeon connecting the kidney to the MP device and start of MP, and preparation of the HMP-kidney for non-accompanied transport to the recipient center. In case of device-related alarms during MP the 24/24h help desk is available to resolve them.

Based on the legally required European tender, Organ Recovery Systems (Diegem, Belgium) was selected as MP company together with the organization of the NPS. Therefore, the NPS is currently organized by Organ Recovery Systems (Diegem, Belgium) and the LifePort Kidney Transporter (®Organ Recovery Systems, Diegem, Belgium) was used for all MP procedures. The device pumps the perfusion fluid through the kidney vasculature in a pulsatile way (pressure-controlled) by arterial cannulation. The pressure was set at 30 mmHg, as most frequently used in clinical setting. Perfusate and kidney are cooled to 2 °C–8 °C using crushed ice outside the sterile container. Based on the results of the COMPARE trial, all kidneys from DCD donors above 50 years old were actively continuously oxygenated during HMP [15]. Oxygenation was realized by uploading the perfusate with oxygen via direct bubble oxygenation (at 0.5 L/min of O_2_ for 15 min) followed by continuous surface oxygenation (at 0.2 L/min of 0_2_ throughout perfusion duration) during HMP [16, 17]. For this, a disposable oxygenation perfusion circuit (LKT-200X, Organ Recovery Systems, Diegem, Belgium) (CE marked since 2021) was used. Active oxygenation in younger DCD donors was possible in study context (ClinicalTrials.gov identifier: NCT05430620) and on surgeon’s request of the recipient center.

Adverse events during total time of MP were real-time monitored by GPS connection and/or reported by the transplant coordinators/surgeons to the MP helpdesk service. In addition, to evaluate the safety and efficacy of the entire HMP service, a feedback evaluation sheet was introduced to report device- and logistic-related adverse events. The role of HMP preservation in the final timing of the transplant procedure (e.g., night to day surgery, emergency to semi-elective) was also polled on this evaluation sheet.

### Study Period

A retrospective analysis was performed of all ECD and DCD donor procedures performed between October 1, 2022, and September 30, 2023, for which the NPS has been activated. Because HMP was considered by the health authorities as standard of care and with the obligation to provide a yearly evaluation by already obligatory outcome data collection on the Eurotransplant Kidney Registry, no ethical approval was necessary and written informed consent for participation was not required, in accordance with the national legislation and institutional requirements.

### Donor and Recipient Demographics

The following donor demographics were collected: deceased donor type (DBD ECD versus DCD), age, body mass index (BMI), arterial hypertension, diabetes, cause of death, last serum creatinine, total donor warm ischemia time (defined in DCD as the time from withdrawal of mechanical ventilation until the start of *in-situ* cold perfusion). IGL-1 (Institut Georges Lopez, Lissieu, France) preservation solution was used in all donors. Standard procurement techniques were used in DBD and DCD donors. DCD donors were classified according to Belgian modified classification of Maastricht in which a category V is a DCD donor after euthanasia [18].

The following recipient demographics were retrospectively collected: age, BMI, dialysis status pretransplant, time on dialysis and number of kidney transplantation).

### Perfusion Parameters During HMP

Renal flow (RF) and resistance (RR) were continuously registered by the MP device. The following device related parameters were collected: RF and RR at 2 min, 60 min and at the end of perfusion and total perfusion time.

### Transplant Outcome

The following parameters were retrospectively collected to evaluate functional graft outcome: anastomosis time during graft implantation, total cold ischemia, primary nonfunction (PNF; defined as the permanent lack of graft function from time of transplantation when a kidney graft was well perfused but never functioned, necessitating dialysis after kidney transplantation), DGF (defined as the need for dialysis during the first week after transplantation excluding dialysis for hyperpotassemia within the first 24 h after transplantation), acute rejection, estimated glomerular filtration rate (eGFR) (calculated by the Chronic Kidney Disease Epidemiology Collaboration (CKD-EPI creatinine equation) at 1 year, graft and death-censored graft- and patient survival at 1 year.

### Impact on National Utilization Rate and Budget Impact Analysis

To evaluate the impact of the introduction of HMP on the national utilization rate of ECD and DCD kidneys procured and transplanted in Belgium, all isolated kidney and combined organ kidney transplantations originated from deceased donors in Belgium performed between October 2017 and September 2024 as registered in the Eurotransplant Database were retrospectively collected and analyzed as well as the impact on donor age and international exchange of kidneys.

In addition, a budget impact analysis for the NIDHI was performed comparing the expected cost of hemodialysis patients on the waiting list (44.932€/year/patient) versus the economy of reducing dialysis cost due to the net increase in the national use of ECD and DCD kidneys with HMP (3.500€/procedure) which would otherwise be rejected by using SCS preservation alone. As the transplantation costs (immunosuppressive drugs, staff time, …) are identical for SCS and HMP (excluding the costs of the conservation method) this was not used for the budget analysis, nor was the relatively small saving in SCS-cost.

### Statistical Analysis

Characteristics of the donor, the recipient and transplant outcome were described by classical descriptive statistics and comparison with historical data was performed by the chi-square test for differences in proportions. A p-value <0.05 was considered statistically significant. Continuous variables are provided as median and interquartile range. Statistical analysis and plots were accomplished with SAS (version 9.4M8) statistical software and Prisms 10.5.0 (Graphpad Software, San Diego, CA, USA).

## Results

### Activation of National Machine Perfusion Service and Donor Characteristics

Between 1 October 2022, and 30 September 2023, the national machine perfusion service was activated for 178 deceased donor procedures with a potential of 356 transplantable kidneys. Donor type, age, comorbidity and serum creatinine are shown in Table 1. In the DCD group 50 (36%), 31 (23%), 46 (33%) and 11 (8%) donors were in the age group of 0–50 years, 51–60 years, 61–70 years and above 71 years old, respectively. For DCD procedures, a rapid canulation of the abdominal aorta and normothermic regional perfusion was performed before kidney procurement in 175 (98%) and 3 (2%), respectively.

**TABLE 1:** Donor characteristics of the 178 deceased donor procedures for which the national machine perfusion service was activated in Belgium between 1 October 2022, and 30 September 2023.

Donor characteristics	178 deceased donor procedures
Type of donor, n (%)
- DBD-ECD	36 (20.2%)
- DCD
• DCDIII	139 (78.1%)
• DCDV	3 (1.7%)
Donor age, yrs
- DBD-ECD	65 (61,25–68,75)
- DCD
• DCDIII	57 (44–65)
• DCDV	54 (33–57)
Donor comorbidity, n (%)
- Arterial hypertension	54 (30%)
- Diabetes mellitus	11 (6%)
Donor last serum creatinine before kidney procurement (mg/dL)	0.63 (0.5–0.76)
Total donor warm ischemia time for DCD donors (min)	12 (6–20)

Abbreviations: DBD, donation after brain death; DCD, donation after circulatory death; ECD, expanded criteria donors; HMP, hypothermic machine perfusion.

### Discarded Kidneys, Preservation Strategy and Transplantation Rate

**FIGURE 1:**
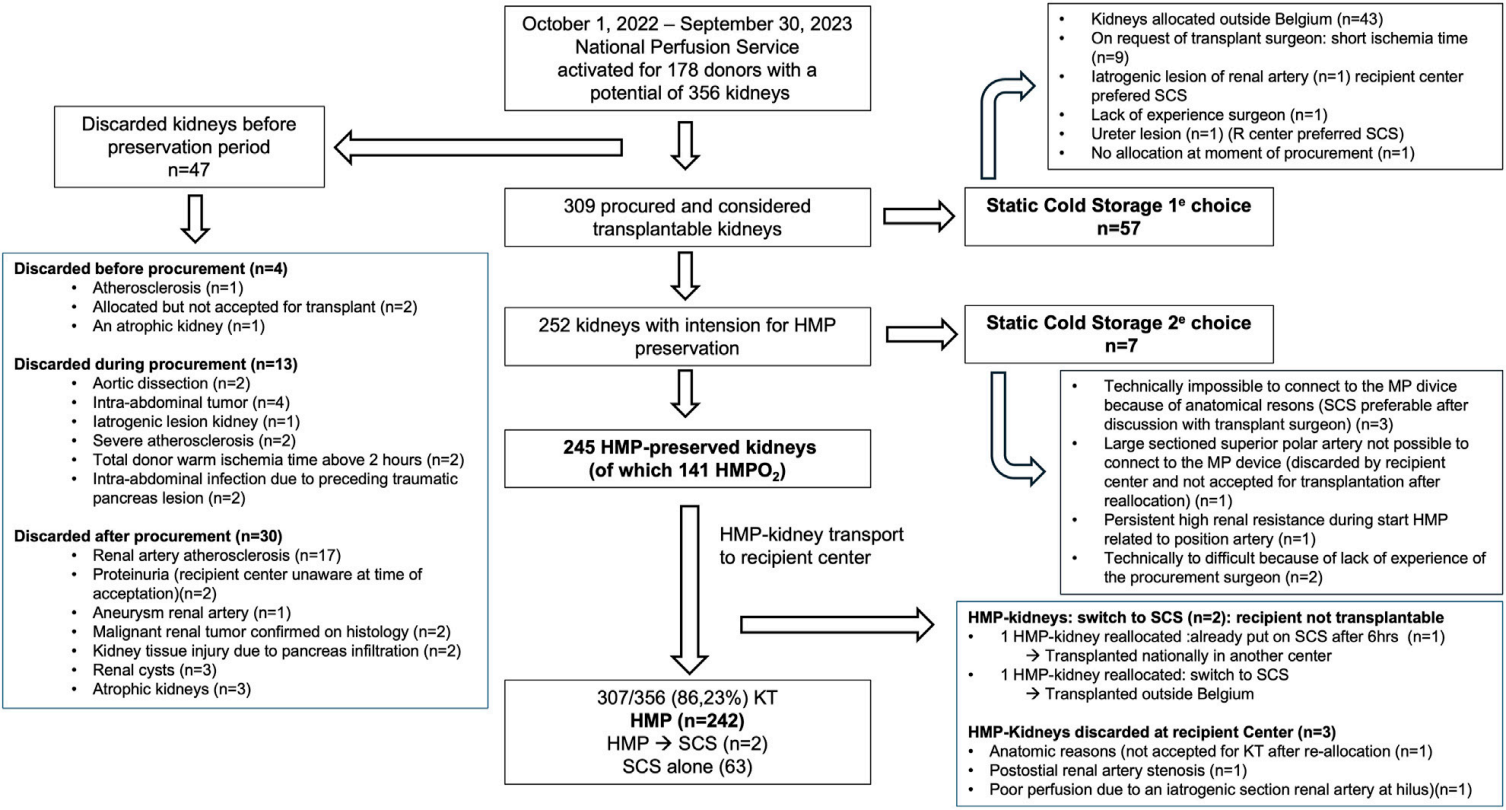
Kidney discard reasons, preservation strategy and transplantation rate of the 356 potential kidneys of the 178 donors for which the national perfusion service was activated between 1 October 2022, and 30 September 2023.

A detailed overview of the kidney discard reasons, preservation strategy and transplantation rate is provided in Figure 1. In summary, forty-seven (13.2%) of the 356 potential donor kidneys were discarded before (n = 4), during (n = 13) and after the procurement (n = 30) because of medical contra-indications for transplantation which resulted in a total of 309 kidneys deemed transplantable. Of them, SCS was used instead of MP in 57 (18.5%) mainly for export outside Belgium since no reimbursement of HMP was foreseen in this setting (n = 43) and on request of the transplant surgeon in case of very short estimated cold ischemia time (n = 9). All these SCS-preserved kidneys were successfully transplanted.

In total, 252 kidneys were intended to be preserved by HMP of which 97% (n = 245) were successfully connected to the MP device. In three percent (n = 7), the procurement surgeon encountered a technical issue when connecting the kidney to the MP device or immediately after start of MP and organ preservation was immediately switched to SCS. Exact reasons are also detailed in Figure 1. Six of these 7 SCS-preserved kidneys were successfully transplanted.

Finally, 245 HMP-kidneys were transported from donor to the recipient center. In a minority of the cases the kidney procurement was in the same center as the transplant procedure. Two of them were reallocated afterwards (according to standard Eurotransplant kidney allocation rules) because the recipients were not transplantable due to unexpected medical reasons. Both kidneys were switched to SCS, awaiting acceptance of the reallocation procedure, and afterwards successfully transplanted, one in Belgium and one abroad. Three of the 245 HMP-kidneys were discarded in the recipient center by the transplant surgeon for the following reasons: anatomic reason (n = 1), postostial renal artery stenosis (n = 1) and poor perfusion (by macroscopic evaluation) during HMP due to iatrogenic section of the renal artery at the hilus (n = 1). Of note, no kidney was discarded based on high renal resistance values.

The transplantation rate of ECD and DCD kidneys during this first year after the implementation of HMP was 86.23% (n = 307) of which 242, 63 and 2 were preserved by continuous HMP, SCS alone, and HMP switched to SCS preservation, respectively.

A retrospective analysis was performed on these 242 isolated HMP-perfused kidney transplantations of which 49 from ECD and 193 from DCD donors. Their donor characteristics are shown in Table 2.

**TABLE 2:** Donor characteristics according to donor type of the 242 patients receiving an HMP-preserved kidney graft in Belgium between 1 October 2022, and 30 September 2023, procured from national ECD and DCD donors.

Donor type	All (ECD + DCD) (n = 242)	DBD ECD (n = 49)	DCD (n = 193)	DCD ≤ 50 years (n = 77)	DCD > 50 years (n = 116)
Donor age, yrs	58 (47–65)	64 (61–68)	55 (43–64)	42 (35–46)	61 (56–67)
Donor sex, male/female, n (%)	134 (55%)/108 (45%)	25 (51%)/24 (49%)	108 (56%)/85 (44%)	52 (68%)/25 (32%)	56 (48%)/60 (52%)
Donor body mass index	25 (23–28)	25 (24–28)	25 (22–29)	24 (21–28)	28 (23–29)
Donor comorbidity, n (%)					
- Arterial hypertension	68 (28%)	25 (51%)	43 (22%)	6 (8%)	37 (32%)
- Diabetes mellitus	15 (6%)	3 (6%)	12 (6%)	0 (0%)	12 (10%)
Donor last serum creatinine before kidney procurement (mg/dL)	0.6 (0.5–0.75)	0.72 (0.6–0.78)	0.57 (0.45–0.71)	0.57 (0.45–0.67)	0.54 (0.44–0.71)
Total donor warm ischemia time for DCD donors (min)	NA	NA	15 (11–21)	13 (11–23)	15 (12–21)

Abbreviations: DBD, donation after brain death; DCD, donation after circulatory death; ECD, expanded criteria donor; NA, not applicable; yrs, years.

### Machine Perfusion Parameters and Adverse Events

Table 3 summarizes the renal resistance, renal flow, total perfusion time and adverse events during MP. The mean renal resistance of the 242 successfully transplanted kidneys was 0.42 (0.32–0.62) mmHg/ml/min, 0.25 (0.20–0.33) mmHg/ml/min and 0.19 (0.14–0.25) mmHg/ml/min, at 2 min, 30 min, and at the end of the perfusion period, respectively. The median total MP time was 5h15 (2h57 – 8h48).

**TABLE 3:** Machine perfusion parameters and adverse events of the 242 HMP-preserved kidneys transplanted in Belgium between 1 October 2022, and 30 September 2023, procured from national ECD and DCD donors.

Machine perfusion parameters	242 HMP-preserved kidneys
Renal resistance, mmHg/mL/min	
- At 2 min	0.42 (0.32–0.62)
- At 30 min	0.25 (0.20–0.33)
- At end of perfusion	0.19 (0.14–0.25)
Renal flow, mL/min	
- At 2 min	60 (41–77)
- At 30 min	87 (72–102)
- At end of perfusion	105 (84–127)
Total perfusion time, h:m	5h15 (2h57 - 8h48)
Adverse events during MP	Details
- “Upstream bubble alarm” with stop MP (n = 4)	- Restart MP during transport (n = 2) or at arrival in recipient center (n = 2)
- “Leakage” at perfusion cannule with stop MP during organ transport (n = 1).	- Exploration in recipient center during bench preparation: due to a small aorta patch (pediatric donor) the cannule was not fixed tight enough
- “High resistance alarm” (n = 3)	- Movement during transport (n = 1) or during delivery of the kidney in the recipient center (n = 1): MP manually restarted
- “High pressure alarm” without interruption of MP (n = 1)	- Sudden peak (n = 1) of resistance spontaneously normalized without interruption of MP
- “High pressure alarm” with interruption of MP (n = 1)	- Unknown cause (probably transport-related) but alarm resolved by decreasing the pressure from 30 to 20 mmHg
	- Probably related to a kinking of the artery during transport: restart MP during transport

Abbreviations: DCD, donation after circulatory death; ECD, expanded criteria donors; HMP, hypothermic machine perfusion.

In total 10 adverse events during MP were recorded/reported. Details and action of handling to restart HMP are also illustrated in Table 3.

### Recipient and Transplant Characteristics

The recipient and transplant characteristics are detailed in Table 4.

**TABLE 4:** Recipient and transplant characteristics of the 242 patients receiving an HMP-preserved kidney graft and transplanted in Belgium between 1 October 2022, and 30 September 2023, procured from national ECD and DCD donors.

Recipient and transplant characteristics	HMP(O_2_) (n = 242)
Recipient characteristics	
Age, yrs	61 (53–68)
Body Mass index (BMI), n	26 (23–29)
Preemptive kidney transplant, n (%)	15 (6.20)
Time on dialysis, days	1,044 (617–1,497)
Number of kidney transplantation, n (%)	
- First kidney transplants	227 (93.8)
- Second kidney transplants	15 (6.2)
Transplant characteristics	
Anastomosis time, min	30 (25–35)
Total cold ischemia time	8h33 (5h17–12h45)

Abbreviations: DCD, donation after circulatory death; ECD, expanded criteria donors; HMP, hypothermic machine perfusion; HMPO_2_, oxygenated HMP.

The anastomosis and total cold ischemia time were 30 (25–35) min and 8h33 (5h17–12h45), respectively.

### 1-Year Functional Outcome

The 1-year functional outcome, according to donor type, is shown in Table 5. Delayed graft function, median eGFR, organ rejection rate and overall graft-, death-censored graft- and patient survival at 1 year after transplantation for all the 242 ECD and DCD kidneys were 14.35%, 50 mL/min/1.73 m^3^, 10.05%, 96.28% and 98.24%, respectively. The reasons for graft loss (13/242) were primary nonfunction (n = 1), rejection (n = 1), donor-transmitted infection (n = 1), graft infection (n = 1), arterial thrombosis (n = 1), venous thrombosis (n = 2), unknown (n = 2) and patient death (n = 4).

**TABLE 5:** One-year functional outcome of the 242 HMP-preserved kidneys successfully transplanted in Belgium between October 1, 2022, and September 30, 2023, procured from national ECD and DCD donors.

Donor type	All (ECD + DCD)	DBD ECD	DCD	DCD ≤ 50 years	DCD > 50 years
Type of kidney preservation (HMP, HMPO_2_)	HMP(O_2_) (n = 242)	HMP (n = 49)	HMP(O_2_) (n = 193)	HMP(O_2_)^$^ (n = 77)	HMPO_2_ (n = 116)
Delayed graft function (%)	14.35	9.14	16.67	9.46	19.64
Primary nonfunction (%)	0.4	0	0	0	0.9
eGFR @1 year (ml/min/1.73 m^2^)	50 (41–66)	49 (38–66)	50 (42–68)	61 (48–78)	48 (40–61)
Organ rejection @1 year (%)	10.05	2.33	12.82	8.96	14.00
Overall graft survival @1 year (%)	94.63	95.91	94.8	97.40	92.24
Death-censored graft survival @1 year (%)	96.28	100	94.34	97.40	93.97
Patient survival @1 year (%)	98.34	95.91	98.96	100	98.28

Abbreviations: DBD, donation after brain death; DCD, donation after circulatory death; ECD, expanded criteria donor; eGFR, estimated glomerular filtration rate; HMP, hypothermic machine perfusion; HMPO_2_, oxygenated hypothermic machine perfusion; y, year; yrs, years.

^a^ HMP n = 63; HMPO2 n = 14.

### Impact of HMP on the National Utilization Rate

**FIGURE 2:**
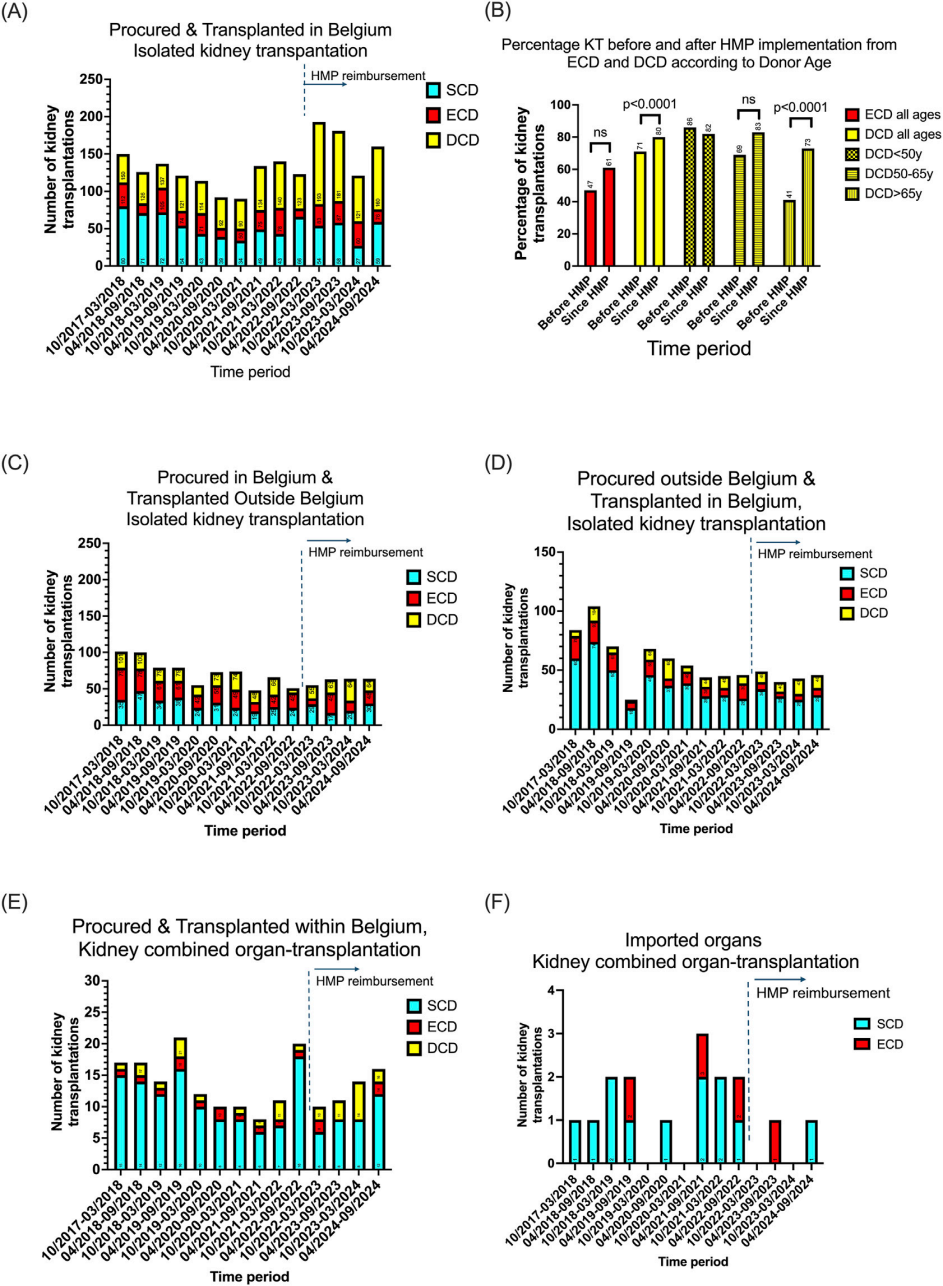
The national implementation of HMP on kidney utilization rate in Belgium between October 2017 and March 2024 resulted in an increase of total number of kidney transplantations originated from Belgian DCD and ECD donors regardless of donor age **(A)**. The observed increase was more pronounced in the donor age groups between 50 and 65 years old and above 65 years, but not for DCD kidneys with donor age below the age of 50 **(B)**. The numbers of kidney transplantations from exported **(C)** and imported kidneys in Belgium **(D)** did not change after the introduction of HMP. A mild increase of kidney combined organ-transplantation from especially ECD and DCD was observed **(E)**. Import in Belgium of a kidney for combined organ transplantation is rare **(F)**.

Compared to historical data from October 2017 and March 2024, the national implementation of HMP resulted in a significant increase of the total number of kidney transplantations originating from Belgian DCD donors, regardless of donor age, from 90 before HMP implementation until 175 per year afterwards (p < 0.0001). ECD Kidney transplantation increased from 45 before, up to 54 per year after the implementation of HMP (p = 0.2965) (Figure 2A). The observed increase was more pronounced in the DCD donors above 50 years old and only significant for donor age above 65 years old, but not for DCD kidneys with donor age below the age of 50 (Figure 2B). The numbers of kidney transplantations from exported and imported kidneys in Belgium did not change after the introduction of HMP, Figures 2C,D, respectively. A mild increase of kidney combined organ-transplantation especially from ECD and DCD is illustrated in Figure 2E. As illustrated in Figure 2F, import in Belgium of a kidney for combined organ transplantation is rare.

### Budget Impact Analysis of HMP Implementation

Based on the historical numbers of DCD and ECD kidney transplantations in Belgium as detailed in the previous paragraph, the routine use of HMP for DCD and ECD kidney resulted in a potential more or less 99 national extra DCD and ECD kidney per year as compared to the historical standard SCS preservation. Therefore, based on an estimated 240 procedures per year, the saving of dialysis costs for the health insurance was −3.592.265€ per year with a half-cycle correction (i.e., reducing the annual saving by 50% in order to correct for the timing effect of the transplant procedure during the year) of 1.376.132€ per year (based on costs of HMP (3.500€/procedure) and dialysis (44.932€/year/patient). The profit percentage accounts 163.8% versus the costs of machine perfusion.

## Discussion

This national cohort study demonstrates that the introduction of a centralized HMP service for all ECD and DCD donor in Belgium resulted in excellent one-year transplant outcomes. The implementation led to a substantial increase in the national utilization rate of ECD and DCD kidneys without impacting export rates. Furthermore, the approach proved to be highly cost-effective, with estimated annual savings of €3.59 million in dialysis-related costs.

**FIGURE 3:**
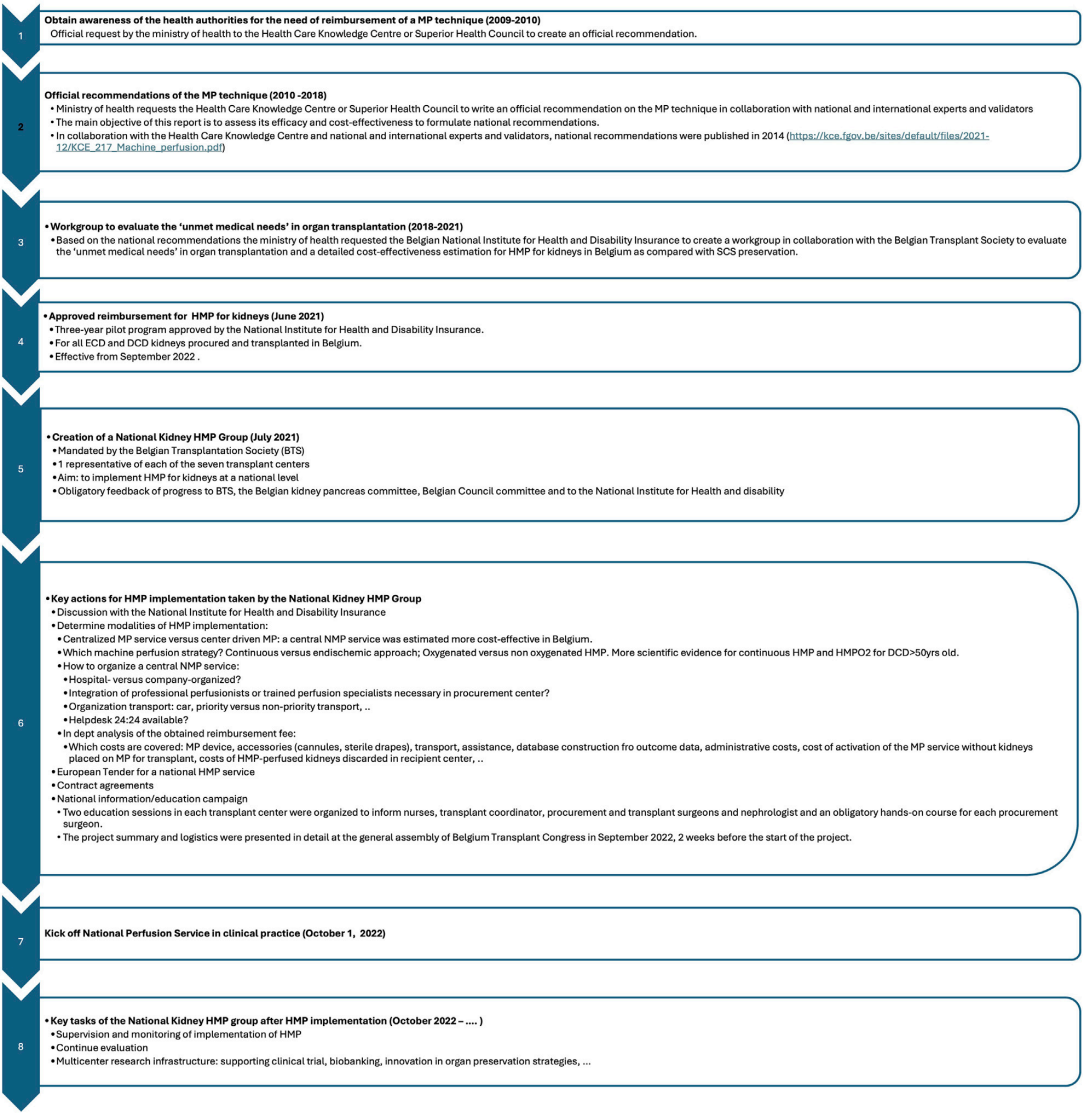
Flow chart with the key elements such as stakeholder alignment, reimbursement mechanisms, and operational workflows to realize a successful national implementation for hypothermic machine perfusion preservation for kidneys based on the Belgian experience.

Despite the well-documented advantages of HMP over SCS, including lower rates of DGF and improved graft survival, many countries have struggled to implement HMP at scale due to reimbursement and logistic barriers [1–11, 15]. The study of Brat A et al is the only study reporting that continuous nonoxygenated HMP preservation as a nationwide standard mode of preservation in the Netherlands of deceased donor kidneys is feasible and safe and resulted in an increase of DCD kidney transplantation, preemptive transplantation and retransplants as well as a significant reduction of DGF as compared with a historical cohort group [19]. The Belgian experience illustrates how national-level implementation is feasible through close collaboration between stakeholders, coordinated logistics, and proactive policy support. Key elements for success, such as stakeholder alignment, reimbursement mechanisms, and operational workflows, are summarized in Figure 3. The creation of a National Kidney HMP group was essential and pivotal for its implementation. Therefore, countries or regions who want to embark on a HMP program should take this as a key element for success. Another critical component of the implementation was the real-time communication and mutual trust between procurement and transplant teams. In cases of anatomical anomalies or procurement complications, transplant centers were directly consulted to decide on preservation strategy, contributing to a low device failure or conversion rate (3%). This approach also limited unnecessary use of perfusion kits, optimizing budget.

The one-year functional outcomes in this cohort are in line with, and in some cases exceed, results reported in major randomized trials such as the COMPARE trial and the Eurotransplant MP trial [2, 15]. The overall DGF incidence in our cohort was 14.35%, and 19.64% among DCD donors older than 50 years—a notable improvement compared to 36% in the COMPARE trial’s HMP groups. The use of continuous HMP from procurement to implantation, combined with short ischemia times, low associated donor comorbidity and uniform procedural standards across centers (e.g., standardisation of DCD procedures across all Belgian center (therapy withdrawal in OR, rapid procurement technique)), may explain this favourable outcome. These parameters reflecting the Belgian situation, and therefore, the expected results of a nationwide implementation of HMP in other countries will be influenced by national geography, local practice in kidney acceptance in case of high donor-associated comorbidity and local procurement strategies. However, our results are also in line with a *post hoc* analysis of the Eurotransplant MP trial demonstrating remarkably lower rates of DGF in kidneys transplanted after a very short cold ischemia time [11].

Since September 2022, oxygenated HMP (HMPO_2_) has been applied to all DCD kidneys from donors aged >50 years, based on COMPARE trial findings. The rationale for HMPO_2_ lies in maintaining aerobic metabolism and limiting ischemia–reperfusion injury by preventing accumulation of harmful metabolites [20–22]. While current evidence remains heterogeneous, recent studies and meta-analyses suggest that HMPO_2_ may reduce severe adverse events and may be particularly beneficial in DCD kidneys and in continuous, rather than end-of-preservation, application (Table 6) [15, 23–28]. However, large-scale prospective data are still needed to conclusively define the added value of oxygenation.

**TABLE 6:** Human clinical studies and series using oxygenated hypothermic machine perfusion preservation for kidneys between 2019 and June 2025.

Author, (year)	Study design	Population	DGF, n (%)	PFN, n (%)	eGFR (mL/min)	Graft survival (%)	Rejection, n (%)
Meister (2020)	RCT III Kidney Assist™	ECD: HMPO_2_ n = 15 SCS n = 30 p-value	8 (53.0%) 10 (33.0%) 0.197	1 (7.0%) 0 (0.0%) 0.333	@6 months 32 ± 14* 38 ± 17* 0.276	@6 months 93.0% 100.0% 0.333	NA NA
Ravaioli (2020)	Non-RCT II	ECD: cHMPO2 n = 10 SCS n = 30 p-value	2 (20.0%) 12 (40.0%) 0.607	0 (0.0%) 1 (3.3%) 0.948	NA NA	@1y 100.0% 93.3% 0.894	@6 months 1 (10.0%) 2 (6.7%)
Jochmans (2020)	RCT III Kidney Assist™	DCD >50 years: cHMPO2 n = 83 cHMP n = 83 p-value	38 (36.0%) 38 (36.0%) 0.990	3 (3.0%) 5 (5.0%) 0.480	@1y 50 ± 19 47 ± 17 0.120	@1y 97.0% 90.0% 0.028	@1y 15 (14.0%) 27 (26.0%) 0.040
Husen (2021)	RCT III Kidney Assist™	ECD: SCS + eHMPO2 n = 127 SCS n = 135 p-value	30 (23.6%) 38 (28.1%) 0.400	8 (6.3%) 8 (5.9%) 0.900	@1y 40 ± 14 41 ± 17 0.630	@1y 92.1% 93.3% 0.630	@1y 23 (18.1%) 18 (13.3%) 0.290
Pravisani (2022)	Retrospective cohort study Waves® LifePort® kidney transporter	DBD: SCS + eHMPO2 n = 51 (Waves®) SCS + eHMP n = 52 (LifePort®) p-value	11 (21.5%) 13 (25.0%) 0.580	0 (0.0%) 0 (0.0%) 1.000	sCreat (mg/dL) 1.27 (1.09–1.67)* 1.40 (1.00–1.78)* 0.319	NA NA	6 (11.7%) 4 (7.7%) 0.520
Dajti (2025)	RCT Vitasmart®	ECD: SCS + eHMPO2 (min 2h) n = 54 SCS n = 55 p-value	31 (57.4%) 37 (67.3%) 0.300	3 (5.5%) 4 (7.3%) 0.700	@1y 50 ± 10 45 ± 14 0.200	@1y 83.6% 88.9% 0.400	@1y 4 (7.3%) 8 (14.8%) 0.200
Darius (2025)	Retrospective belgian cohort LifePort® kidney transporter	DCD ≥50 years: cHMPO_2_ n = 116	22 (19.7%)	1 (0.9%)	@1y 50 (14.3–94.2)*	@1y 92.2%	@1y 14 (12.0%)

Abbreviations: cHMP, continuous hypothermic machine perfusion; cHMPO2, continuous oxygenated hypothermic machine perfusion; DBD, donation after brain death; DCD, donation after circulatory death; DGF, delayed graft function; ECD, expanded criteria donors; eGFR, estimated glomerular filtration rate; eHMPO2, endischemic oxygenated hypothermic machine perfusion; HMP, hypothermic machine perfusion, HMPO2, oxygenated hypothermic machine perfusion; SCS, static cold storage.

Data are presented as mean± range or mean (min-max) if indicated with*.

Notably, in our cohort, no kidney was discarded solely due to high RR during HMP. While elevated RR is frequently cited as a reason for organ discard, especially in the US where 33% of pumped kidneys are not transplanted, our results support a more nuanced interpretation [29–33]. One kidney with persistently high RR due to the position of the renal artery was successfully transplanted after switching to SCS at the surgeon’s discretion. This underscores that RR should not be used as a stand-alone viability criterion and must be interpreted within a clinical context. According to a recent meta-analysis, current evidence does not support the use of isolated RR-thresholds for organ acceptance [29].

Although HMP implementation significantly increased the number of transplanted ECD/DCD kidneys, the total national kidney transplant volume did not rise proportionally. This reflects a broader trend of declining donor availability and increasing waiting lists and waiting list mortality. In addition, the overlapping COVID-19 period and the increased confidence in these kidneys because of the use of HMP during the study period are important parameters that might have influenced the utilization rate in addition to the HMP reimbursement itself. Nonetheless, the increased utilization of higher-risk organs maintained overall transplant activity, which may otherwise have declined. The excellent outcomes observed should help increase clinician confidence in accepting higher risk organs, potentially improving acceptance rates in the future.

The economic impact of HMP is supported by earlier evaluations of the Eurotransplant MP trial, which also showed cost savings through reduced DGF incidence [12, 13]. In our budget impact analysis—comparing the expected dialysis costs of patients on the waiting list (€44.932/year/patient) versus the economy of reducing dialysis cost due to the net increase in national use of ECD and DCD kidneys with HMP (€3.500/procedure) — the increase in transplant activity translated to an estimated 99 additional national kidney transplants per year, yielding substantial healthcare savings of €3.59 million per year.

We acknowledge the following limitations of this manuscript: 1) the lack of a historical control or propensity matched group, 2) the modified DGF definition that excludes one dialysis session within the 24 h after transplantation could slightly underestimate the DGF rate as compared to studies including this in the DGF definition, 3) the retrospective design, 4) the short time follow-up, 5) the limited generalizability to other health systems because of the specific organizational structure in Belgium, and 6) the absence of a real cost-effectiveness analysis.

Based on these findings, the Belgian NIHDI approved a five-year extension of the HMP reimbursement scheme (2025–2030), considering now HMP as standard of care for kidney preservation. The revised criteria now also include HMP for kidney combined-organ transplants, DBD donors <50 years with peak creatinine >1.5 mg/dL, and cross-border HMPO_2_ import from the Netherlands. Implementation of cross-border HMP exchange will require harmonization of logistics, regulation, and reimbursement across Eurotransplant countries.

Finally, the national HMP platform offers a unique opportunity to evolve into a multicentre research infrastructure supporting clinical trials, biobanking, and innovation in organ preservation strategies. Future research will focus on refining viability assessment tools, comparing continuous versus end-HMPO_2_, and long-term cost-effectiveness in real-world settings. Experience gained from this national effort will help to implement MP of other organs.

## Data Availability

The raw data supporting the conclusions of this article will be made available by the authors, without undue reservation.
